# Gender Parity Analysis of the Editorial Boards of Influential Dermatology Journals: Cross-Sectional Study

**DOI:** 10.2196/40819

**Published:** 2024-05-21

**Authors:** Mindy D Szeto, Torunn E Sivesind, Lori S Kim, Katie A O’Connell, Kathryn A Sprague, Yvonne Nong, Daniel M Strock, Annie L Cao, Jieying Wu, Lauren M Toledo, Sophia M Wolfe, Wyatt Boothby-Shoemaker, Robert P Dellavalle

**Affiliations:** 1 Department of Dermatology University of Minnesota Medical School Minneapolis, MN United States; 2 Department of Dermatology University of Colorado Anschutz Medical Campus Aurora, CO United States; 3 Department of Dermatology Boston University School of Medicine Boston, MA United States; 4 Department of Dermatology Vanderbilt University Medical Center Nashville, TN United States; 5 Chicago College of Osteopathic Medicine Downers Grove, IL United States; 6 Michigan State University College of Human Medicine Flint, MI United States; 7 Eastern Virginia Medical School Norfolk, VA United States; 8 College of Osteopathic Medicine Rocky Vista University Parker, CO United States; 9 Lake Erie College of Osteopathic Medicine Bradenton, FL United States; 10 Department of Dermatology Henry Ford Hospital Detroit, MI United States

**Keywords:** diversity, equity, inclusion, editors, journals, publications, editorial board, women, gender, underrepresentation

## Abstract

This study underscores the persistent underrepresentation of women in academic dermatology leadership positions by examining the gender composition of editorial boards across top dermatology journals, emphasizing the urgent need for proactive strategies to promote diversity, equity, and inclusion.

## Introduction

Women continue to be underrepresented in academic leadership positions, especially in dermatology [1]. Although women account for more than half of all board-certified dermatologists in the United States, academic dermatology leadership roles, such as department chair and fellowship director positions, remain disproportionately occupied by men [2]. This inequity extends to medical journals, with substantial gender gaps reported in editorial board composition across multiple specialties; previously published data from 2018 suggested that women accounted for the minority of dermatology editors in all positions [1]. To provide an evaluation of current trends, the composition of dermatology editorial boards by gender was assessed in 2021, making comparisons among highly indexed dermatology journals.

## Methods

The top 20 most impactful dermatology journals by the 2020 *h*-index were identified on Scimago [3]. Journal editorial board websites were searched in November 2021 for lists of editor names and roles, and journal-defined editorial board members were identified and tabulated. Binary (women vs men) gender estimation by author first name was performed with Gender API [4], a popular gender inference service based on querying large multifactorial databases and name repositories. Estimations were corroborated by web-based searches of professional photographs and biographies by 2 independent researchers, with in-depth discussion and consensus meetings to resolve discrepancies.

## Results

**Figure 1:**
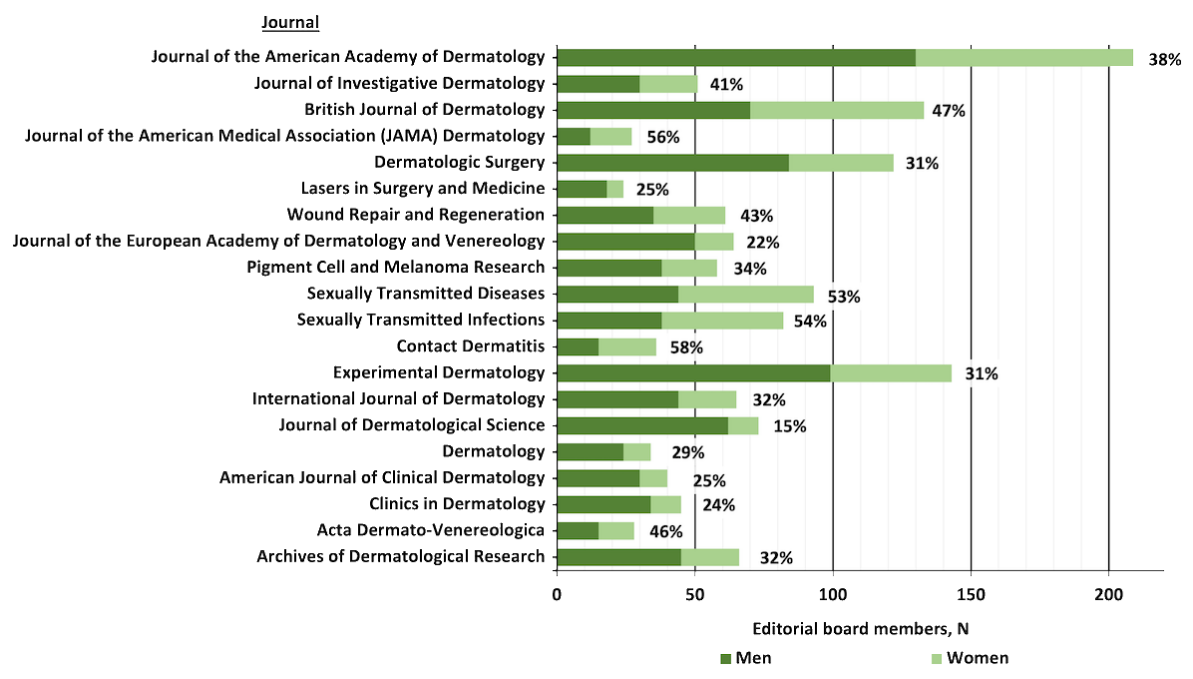
Numbers of men and women on editorial boards for the top 20 dermatology journals by h-index. Percentages of women editorial board members are indicated.

Editorial board membership averaged 37% (SD 12%) women, with a median of 33% (IQR 18%) women across the journals analyzed (Figure 1 and Table 1). The *Journal of Dermatological Science* (11/73, 15%) and *Journal of the European Academy of Dermatology and Venereology* (14/64, 22%) had the lowest proportions of women editors, whereas *Contact Dermatitis* (21/36, 58%), *Sexually Transmitted Infections* (44/82, 54%), and *Sexually Transmitted Diseases* (49/93, 53%) had among the highest. The editorial board of *Journal of the American Medical Association (JAMA) Dermatology* was observed to be 56% (15/27) women after excluding International Advisory Committee members. Of the 20 journals, only 5 (25%) had women editors-in-chief.

**Table 1:** Women editorial board members and editors-in-chief for the top 20 dermatology journals by the 2020 h-index.

Dermatology journal		*h*-index rank	*h*-index in 2020	Editorial board members, N	Women, n (%)	Woman editor-in-chief
*Journal of the American Academy of Dermatology*		1	208	209	79 (38)	No
*Journal of Investigative Dermatology*		2	201	51	21 (41)	No
*British Journal of Dermatology*		3	179	133	63 (47)	No
*JAMA^a^ Dermatology^b^*		4	166	27	15 (56)	Yes
*Dermatologic Surgery*		5	125	122	38 (31)	No
*Lasers in Surgery and Medicine*		6	112	24	6 (25)	No
*Wound Repair and Regeneration*		7	109	61	26 (43)	No
*Journal of the European Academy of Dermatology and Venereology*		8	107	64	14 (22)	No
*Pigment Cell and Melanoma Research*		9	105	58	20 (34)	No
*Sexually Transmitted Diseases*		10	105	93	49 (53)	No
*Sexually Transmitted Infections*		11	98	82	44 (54)	Yes
*Contact Dermatitis*		12	96	36	21 (58)	Yes
*Experimental Dermatology*		13	96	143	44 (31)	No
*International Journal of Dermatology*		14	93	65	21 (32)	Yes
*Journal of Dermatological Science*		15	93	73	11 (15)	No
*Dermatology*		16	92	34	10 (29)	No
*American Journal of Clinical Dermatology*		17	89	40	10 (25)	Yes
*Clinics in Dermatology*		18	88	45	11 (24)	No
*Acta Dermato-Venereologica*		19	83	28	13 (46)	No
*Archives of Dermatological Research*		20	80	66	21 (32)	No
**All journals**						
	Total	—^c^	—	1454	537 (37)	5/20 (25)^d^
	Mean (SD)	—	—	73 (47)	37 (12)	—
	Median (IQR)	—	—	63 (46)	33 (18)	—

^a^*JAMA: Journal of the American Medical Association*.

^b^*JAMA Dermatology*’s editorial board was observed to be 36% (19/53) women when including International Advisory Committee Members.

^c^Not applicable.

^d^Reported as n/N (%).

## Discussion

Our findings suggest that an underrepresentation of women on dermatology editorial boards concerningly persists across multiple top journals, recapitulating earlier findings by Lobl and colleagues [1] while highlighting potential ongoing challenges in addressing gender disparities within editorial boards. However, limitations of our study include reliance on high-throughput software examining first names only and estimating binary gender, which may lead to misclassification and lacks acknowledgment of individuals identifying as nonbinary or transgender. Indeed, it has been recognized that Gender API may not be accurate when performing estimations on first names considered to be gender neutral [4]. Future work analyzing self-reported sex and gender identity to ensure true concordance with the individual’s identity is needed.

Abating the gender gap among editorial boards may improve the editorial review process and the diversity of perspectives offered, along with expanding the use of inclusive language and encouraging diverse author representation. Editors-in-chief and academic journal leadership should evaluate board member recruitment with the goal of gender parity, where having 50% women on editorial boards could more accurately represent the dermatology workforce [1]. Furthermore, those serving in senior editor positions may wield considerable influence over the journal and editorial procedures, emphasizing the need for a careful and nuanced approach to fostering overall inclusivity. Subsequent analysis by editor roles, credentials, backgrounds, and experience across different journals may assist with driving meaningful change. As part of *JMIR Dermatology*’s commitment to diversity, equity, and inclusion (DEI) in the publication and peer-review process, a recent editorial uncovered additional areas for improvement in DEI [5]. Very few dermatology journals explicitly include statements about DEI, have DEI-dedicated editorial board members, or present any information about how the peer-review process ensures DEI. Clear commitments and mission statements from journals could assist with formalizing processes and bolstering transparency. *JMIR Dermatology* has now invited >50% women dermatologists to its editorial board [6]. If the journal’s goals are not ultimately reached, conducting investigations into the reasons underlying lower acceptances among applications from women will be important [6]. Given current data trends, proactive strategies such as these are urgently needed to recruit, promote, and retain women dermatologists in academic settings. Regular monitoring and assessment can help identify foci for improvement and demand accountability. Thus, intentional work to establish expanded frameworks, criteria, and recommending actionable strategies across journals will be a crucial component of broadening DEI and presents a worthwhile goal for further research.

## Abbreviations

DEI: diversity, equity, and inclusion
JAMA: Journal of the American Medical Association
